# Circular RNAs and Their Role in Male Infertility: A Systematic Review

**DOI:** 10.3390/biom13071046

**Published:** 2023-06-27

**Authors:** Maria-Anna Kyrgiafini, Zissis Mamuris

**Affiliations:** Laboratory of Genetics, Comparative and Evolutionary Biology, Department of Biochemistry and Biotechnology, University of Thessaly, Viopolis Mezourlo, 41500 Larissa, Greece

**Keywords:** circular RNAs, male infertility, azoospermia, biomarkers

## Abstract

Male infertility is a global health problem that is on the rise. Today, many noncoding RNAs (ncRNAs) are associated with male infertility. Circular RNAs (circRNAs) have recently drawn attention, but a comprehensive understanding of the role of circRNAs in male infertility is limited. This systematic review investigates the differential expression of circRNAs in male infertility or circRNAs that could serve as candidate biomarkers. The PRISMA guidelines were used to search PubMed and Web of Science on 11 January 2023. Inclusion criteria were human participants, experimental studies aiming to associate circRNAs with male infertility reporting differentially expressed circRNAs, and the English language. A total of 156 articles were found, and after the screening and eligibility stages, 13 studies were included in the final sample. Many circRNAs are deregulated in male infertility, and their interactions with miRNAs play an important role in affecting cellular processes and pathways. CircRNAs could also be used as biomarkers to screen patients before sperm retrieval. However, most studies focus on the role of circRNAs in azoospermia, and there is a knowledge gap regarding other subtypes of male infertility. Future research is needed to explore the exact mechanism of action of circRNAs and investigate their use as biomarkers.

## 1. Introduction

Male infertility is a global health problem that affects approximately 7% of the male population [1]. It is a complex pathological condition that involves environmental and genetic factors [2]. More importantly, however, over the past 40 years, sperm counts have decreased by half, and this decline is characterized as alarming [3]. Overall, studies indicate that infertility is on the rise, and one in six couples trying to conceive worldwide is diagnosed as infertile [4]. The male factor also contributes to 40–50% of all cases of infertility [5]. Therefore, there is a growing need for a better understanding of the molecular mechanisms involved in male infertility, leading to new approaches for its prevention, diagnosis, and treatment [6]. 

In recent years, with the development of high-throughput sequencing technologies, non-coding RNAs (ncRNAs), including long non-coding RNAs (lncRNAs), circular (circRNAs), and microRNAs (miRNAs), have emerged as important regulators in the pathogenesis of male infertility, and it is also suggested that many of these molecules have the potential to be used as biomarkers [7,8]. Previous reviews have extensively discussed the expression profiles and regulatory functions in male infertility [9,10]; therefore, these topics are not discussed here. 

**Figure 1:**
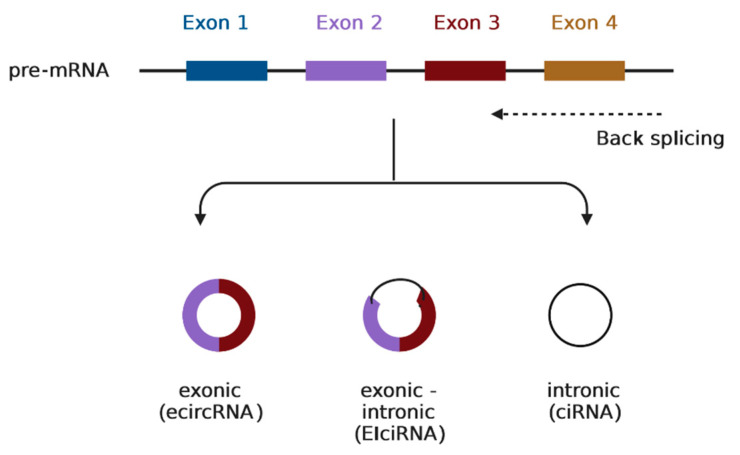
The three subcategories of circRNAs and their formation through back-splicing. Image created with biorender.com.

Circular RNAs (circRNAs) are single-stranded, continuous-loop RNA transcripts that are covalently closed. In general, precursor mRNAs (pre-mRNAs) are transcribed by RNA polymerase II (Pol II), and then they undergo a process called “canonical splicing” that is catalyzed by the spliceosomal machinery and involves the splicing of introns and ligation of exons for the formation of a linear RNA transcript (i.e., mature messenger RNA (mRNA)) with 5′–3′ polarity. However, these canonical spliceosome-mediated pre-mRNAs can also undergo a process called “back splicing” and form circular RNAs in this way. “Back-splicing” is a type of alternative splicing in which the 3′ end of an exon binds to the 5′ end of its own or an upstream exon via a 3′,5′-phosphodiester link, generating a closed structure with a back-splicing junction site [11,12,13]. Due to this structure, they are relatively stable and less susceptible to degradation by Ribonuclease R (RNase R) [14]. RNase R is an exoribonuclease that has the ability to degrade structured RNA molecules such as mRNAs or ribosomal RNA (rRNA) [15]. As circRNAs may arise from exons or introns, they are classified into three main subtypes of circRNAs: exonic (ecircRNAs), intronic (ciRNAs), and exonic-intronic (EIciRNAs) [16] (Figure 1). Furthermore, circRNAs exert their function through various mechanisms [13]. Among others, some studies demonstrate that they can act as miRNA sponges or competing endogenous RNAs (ceRNAs) and upregulate the miRNA target genes, contributing to gene regulation at the post-transcriptional level [17]. In addition, they have complex interactions with several proteins [13]. They can serve as protein sponges or decoys and affect their function [18]. They can also regulate parental gene expression through diverse mechanisms, such as translational modulation and post-translational modification [19]. Finally, their high abundance, stability, and evolutionary conservation between species are some of their intriguing characteristics [14,20], and growing evidence suggests their involvement in many diseases, such as cardiovascular disease, diabetes, cancer, etc. [11]. 

Regarding reproduction, these molecules are gradually gaining the attention of researchers, but despite several sporadic reports about their expression patterns and their potential role in processes associated with fertility, the research on circRNAs in this field is still in its infancy. Many studies also suggest that circRNAs may function as potential biomarkers or therapeutic and pharmaceutical targets in reproduction research [21]. However, it is not clear exactly how circRNAs contribute to male infertility. Most of the data have been obtained mainly from experiments in cell lines and animal models, and limited information is available on whether these results are validated in humans. There are many heterogeneous studies reporting findings on circRNAs and male infertility, but to our knowledge, there are no systematic reviews summarizing the circRNAs that have been associated with male infertility and its specific subtypes or that could serve as biomarkers. A comparison of the studies focusing on humans could be important to identify similar patterns of expression of circRNAs and circRNAs with a regulatory role in male infertility. 

Therefore, to address this gap and determine how far science has advanced regarding circRNAs and male reproduction, the present systematic review aimed to summarize existing studies on humans about the role of circRNAs in male infertility. With this approach, we aim to provide a comprehensive summary of the circRNAs involved in male infertility, which will be of particular interest to the life science community and could serve as candidate biomarkers or therapeutic targets in the future.

## 2. Materials and Methods

This systematic review of the role of circRNAs in male infertility was conducted following the guidelines of the Preferred Reporting Items for Systematic Reviews and Meta-Analyses (PRISMA) statement [22], using the protocol registered in the International Prospective Register of Systematic Reviews (PROSPERO) (ID: CRD42023415884).

**Figure 2:**
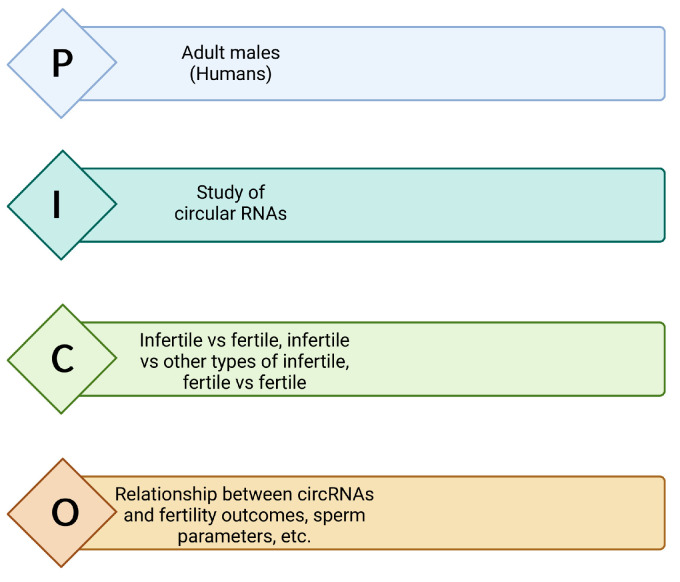
The PICO (P: Population; I: Intervention; C: Comparison; O: Outcome) framework used for this systematic review. Image created with biorender.com.

The PICO (Population, Intervention, Comparison, Outcomes) framework was also used to develop the research question and define our inclusion and exclusion criteria, as presented in Figure 2.

### 2.1. Search Strategy

An extensive search for the available literature about circRNAs and male infertility was conducted on 11 January 2023, using PubMed and the Web of Science databases. 

Following the methodology of two systematic reviews on topics associated with male infertility [9,23], we operationalized our search for the term “male infertility” as follows: #1; “semen” OR “sperm” OR “spermatozoa” OR “oligozoospermia” OR “asthenozoospermia” OR “teratozoospermia” OR “male subfertility” OR “male infertility” OR “spermatogenesis” OR “male infertil*” OR “infertile male*” OR “infertile men” OR “male subfertil*” OR “male steril*” OR “sperm*” OR “seminal”. For the PubMed search, we also used the MeSH (medical subject headings) term “Male Infertility” with the Boolean “OR” to join the searches. The MeSH term included the following terms (subheadings): aspermia, asthenozoospermia, azoospermia, oligospermia, Sertoli Cell-Only syndrome, and teratozoospermia. Similarly, for the term “circular RNAs” and based on the previously validated search of Chen et al. (2020) [24], we operationalized our search as follows: #2; “circular RNA” OR “circular RNAs” OR “circRNA” OR “circRNAs” OR “circ RNA” OR “circ RNAs” OR “ciRNA” OR “ciRNAs”. The final search formula was as follows: #1 AND #2 (a combination of the two terms and all the variant keywords). In addition, for the PubMed search, we applied the title/abstract fields in our search, whereas for the Web of Science search, we used the topic field.

After both searches, the records of all articles were collected, and duplicates were removed using automation tools (Zotero Citation Manager software, https://www.zotero.org/, accessed on 26 June 2023). 

### 2.2. Screening, Eligibility, and Inclusion/Exclusion Criteria

For the screening and eligibility stages, a series of inclusion and exclusion criteria were applied to the articles identified. More specifically, articles were included if they: (i) were written in the English language; (ii) included human subjects (male adults); and/or (iii) were experimental studies reporting differentially expressed (DE) circRNAs and aiming at associating circRNAs with male infertility. Studies were excluded if they were conducted on cell lines, animals, or plants, as well as reviews, meta-analyses, books and book chapters, abstracts of conference presentations, commentary articles, replies, and other types of publications that did not report original data. Furthermore, articles aiming to study other types of ncRNAs, such as piRNAs, miRNAs, etc., were also excluded because the present systematic review focuses on circRNAs and their association with male infertility. The full list of inclusion/exclusion criteria is provided in Table 1.

Based on the above criteria, the articles collected were subsequently screened manually by title, keywords, and abstract to choose those that were appropriate for eligibility. At the eligibility stage, the full text of eligible articles was retrieved to select the final sample of articles included in the present systematic review. These procedural steps were carried out by the first investigator (M.-A.K.), and in case of uncertainty, the second investigator (Z.M.) decided after debate on the final inclusion or exclusion of the studies in the present systematic review.

### 2.3. Data Extraction and Analysis

Information from each article included in the present systematic review was extracted separately by the two investigators (M.-A.K. and Z.M). The two investigators collaborated to resolve any inconsistencies or discrepancies by reviewing the original data of the articles under debate. 

More specifically, the following information was collected from the full text of every included study: (i) title; (ii) reference data (authors, year of publication, country, and journal); (iii) objective(s); (iv) characteristics of the sample (participants, health status, and biological material); (v) experimental design; (vi) circRNAs associated with male infertility and particular subtypes; (vii) genes and pathways affected or mechanism of action of circRNAs; (viii) impact-association with sperm parameters (sperm quality and quantity according to the semen analysis results). 

## 3. Results

### 3.1. Study Selection and Study Characteristics

Our initial search identified 156 articles in total that were imported into the Zotero reference manager. More specifically, 68 of these articles were found from our search in PubMed, and 88 were found from our search in Web of Science. Of these, 63 articles were identified as duplicates and removed, leaving a total of 93 articles for the screening and eligibility stages.

Based on the inclusion and exclusion criteria described in detail above, the 93 articles were manually screened by title, keywords, and abstract for eligibility. Of these, 43 were studies conducted in animals, plants, or cell lines; 20 were articles with non-original data (reviews, letters, etc.); 11 were not relevant to our subject of interest (male infertility or the study of circRNAs); and one was not in the English language, leaving a total of 18 articles for retrieval. More specifically, among the 11 articles excluded as irrelevant, four focused on the utilization of non-coding RNAs, or rRNA genes, as biomarkers in forensic body fluid identification [25,26,27,28]. Five articles examined the role of circRNAs in various types of cancer [29,30,31], as well as their involvement in other pathological conditions such as skeletal muscle atrophy [32] and bronchopulmonary dysplasia [33]. Another article presented a deep learning model that combines convolutional neural network and recurrent neural network architectures to accurately detect splicing sites specific to human circular RNAs using genome sequencing data [34]. Additionally, one article reported a co-analysis system for personal and body fluid identification [35]. We were unable to find the full text of one article; thus, 17 articles were evaluated during the eligibility stage. At the eligibility stage, we excluded four studies that did not report DE circRNAs. Finally, a total of 13 articles remained to be included in the present systematic review [36,37,38,39,40,41,42,43,44,45,46,47]. 

**Figure 3:**
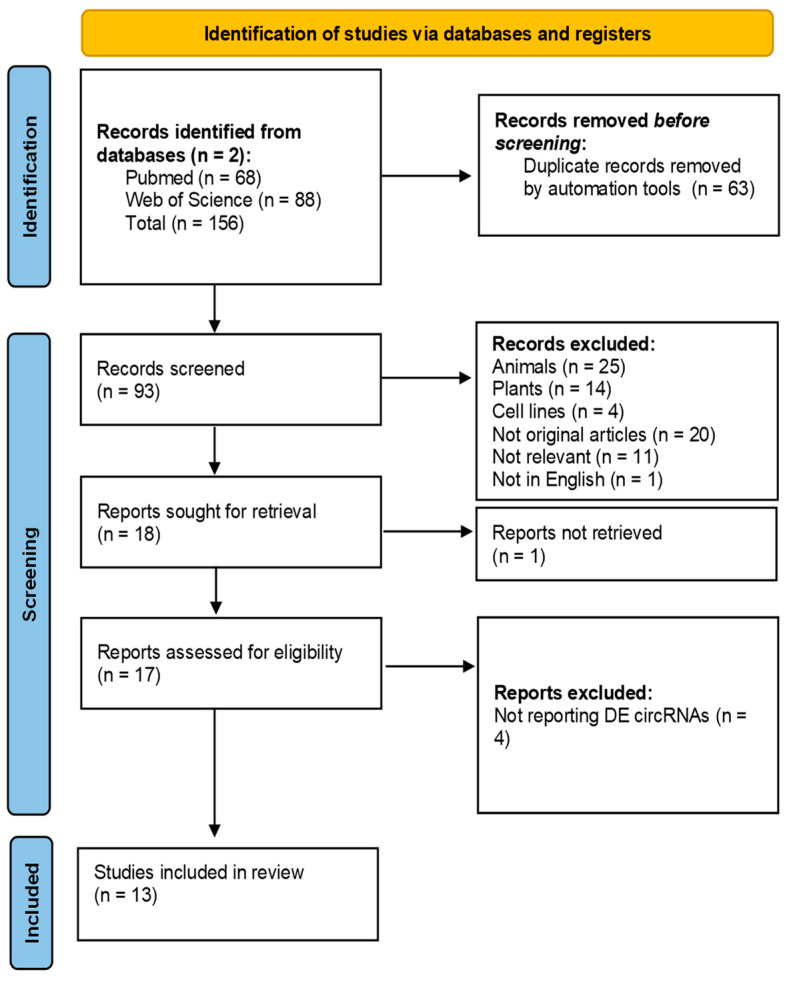
Preferred Reporting Items for Systematic Reviews and Meta-Analyses (PRISMA) diagram of the article selection process used in the present systematic review.

The above steps are presented in detail in the PRISMA flow diagram (Figure 3).

**Figure 4:**
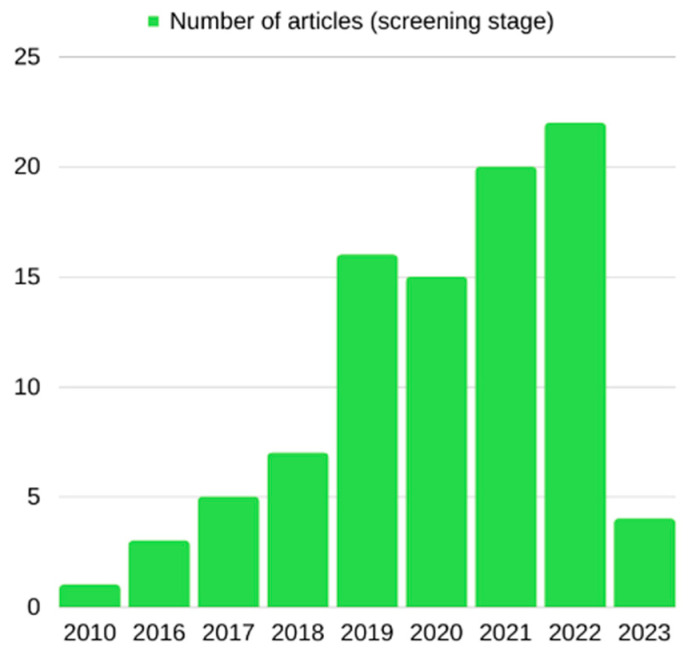
Date of publication of the studies identified in the initial search.

**Figure 5:**
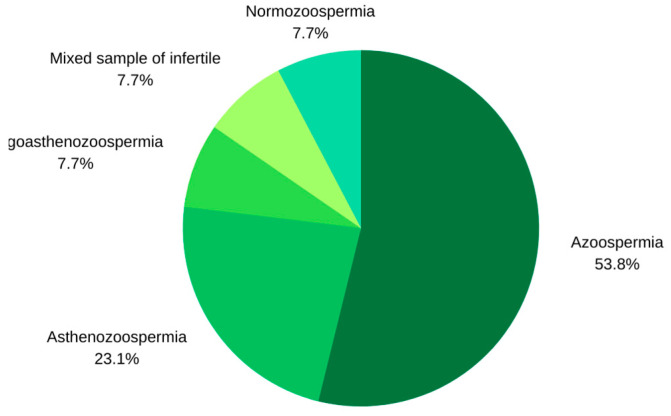
Studies included in the systematic review categorized by subtypes of male infertility.

Regarding study characteristics, of the 93 articles identified from the initial search, only one was published in 2010, but since then, the number of articles published has exponentially grown. In total, three articles were published in 2016, five in 2017, seven in 2018, and a significant increase was observed in 2019, when sixteen articles were published. Subsequently, fifteen articles were published in 2020, 20 in 2021, 22 in 2022, and four articles were published from 1 January until 11 January 2023, as observed in Figure 4. Of the 13 articles included in the analytical sample of the systematic review, the vast majority originated from Asia and, more specifically, from China (9/13, 69.2%), followed distantly by studies from Europe-Italy (3/13, 23,1%), and one study originated from America-USA (1/13, 7.7%). Research on circRNAs in human male infertility was carried out mainly on azoospermia (7/13, 53.8%), followed by research on the role of circRNAs in asthenozoospermia (3/13, 23.1%). One study also explored the contribution of circRNAs to oligoasthenozoospermia (1/13, 7.7%); another study used a mixed sample of infertile men (1/13, 7.7%); and another study was performed on normozoospermic men (1/13, 7.7%) (Figure 5). Finally, the tissues analyzed were testes (n = 6), semen (n = 7), and blood (n = 1). Regarding semen samples, three of them investigated only the seminal plasma and/or the extracellular vesicles found there (n = 3).

Next, we disaggregate the studies included in the present systematic review according to the different subtypes of male infertility. We briefly describe each of these in turn in the next section, starting with azoospermia.

### 3.2. Azoospermia (n = 7)

Seven studies included in the present systematic review focused on the role of circRNAs in azoospermia, the most severe form of male infertility characterized by the complete absence of spermatozoa in the ejaculate [48]. Azoospermia can also be classified into two main types: obstructive azoospermia (OA) and non-obstructive azoospermia (NOA). Three of the studies aimed to identify differentially expressed (DE) circRNAs between testicular tissues of NOA and OA patients. These studies list DE circRNAs between NOA and OA samples but also report some important findings about specific circRNAs. Ge et al. (2019) [43] found that hsa_circ_0023313, a circRNA upregulated in NOA patients, may regulate spermatogenesis by the hsa_circRNA_0023313/miR-372–3p/RAB-24 and/or hsa_circRNA_0023313/miR-373–3p/USP-24 pathways. The hsa_circRNA_0023313 is associated with ubiquitin-protein transferase activity and chromatin binding and increases the expression of target genes through competitive binding with the above miRNAs. This can potentially lead to the pathogenesis of male infertility, as *RAB-24* is an autophagy-related gene, and its differential expression can affect the initiation as well as the progression of spermatogenesis. Bo et al. (2020) [42] draw attention to some other circRNAs that can contribute to NOA pathogenesis through circRNA-miRNA-mRNA interactions. Of interest are hsa_circRNA_072697 and hsa_circRNA_402130. Especially hsa_circRNA_402130 may have a pathological role in male infertility by acting as a “sponge” for the let-7 miRNA family. The let-7 family is an important regulator of stemness by affecting *LIN28A,* and, according to previous studies in mice, it can affect the spermatogenesis process as it can reduce the germ cell pool [49]. Similarly, Zhang et al. (2022) [45] studied DE circRNAs between NOA and OA patients and revealed several pathways that are deregulated through ceRNA networks. The genes targeted by them were found to be associated with axoneme assembly, microtubule-based processes, and cell proliferation. It should be noted that, along the same lines, the two previous studies also reported more deregulated pathways affected by circRNA-miRNA interactions in azoospermia. The DE circRNAs identified in the above studies affect endocytosis, meiosis, the FoxO signaling pathway, ubiquitin-mediated proteolysis, the AMPK signaling pathway, the TNF signaling pathway, and signaling pathways regulating pluripotency of stem cells. Target genes also participate in transcription from the RNA polymerase II promoter, transcriptional misregulation in cancer, transcription, cell migration, etc. [42,43]. In general, most of the biological pathways identified in these studies were considered closely associated with the pathophysiological process of NOA, indicating a crucial role for circRNAs in the disease.

Two studies also aimed to investigate DE circRNAs between NOA and OA patients, but with a particular emphasis on specific histological subtypes of NOA. For example, Zhu et al. (2021) [47] explored the expression profile of circRNAs in patients with Sertoli cell-only syndrome (SCOS). The researchers also investigated the role of the host genes of the DE circRNAs identified, revealing that circRNAs may contribute to SCOS pathogenesis by affecting the testicular immune microenvironment as well as the function and communication of Sertoli cells. They also focused on the circRNA-microRNA interactions of hsa_circRNA_101373, providing evidence that interconnected signaling pathways may be affected, leading to SCOS (e.g., cell differentiation, stem cell maintenance, etc.). With a similar approach, Lv et al. (2020) [38] observed that the expression of hsa_circ_0000116 was significantly higher in testicular samples from NOA patients, but more interestingly, the circRNA was also upregulated in SCOS patients compared to hypospermatogenesis (HS) patients. In an attempt to explore the role of hsa_circ_0000116, Lv et al. (2020) [38] found that the circRNA inhibited the expression of miR-449a in NOA and SCOS patients after comparison with its expression in OA and HS. MiR-449 may participate in regulating autophagy-related genes; therefore, the authors suggest that a hsa_circ_0000116-miR-449-autophagy-related ceRNA network might be involved in the pathogenesis of NOA, as hsa_circ_0000116 may be an inhibitor of spermatogenesis by suppressing miR-449 activity and increasing the expression of autophagy-related genes. Finally, the authors report a finding with potential clinical application since high levels of hsa_circ_0000116 were also associated with a low rate of successful testicular sperm retrieval, indicating that this circRNA could be used as a biomarker for the screening of patients undergoing sperm retrieval. 

A different approach was used by Liu et al. (2020) [44]. The researchers investigated the expression of a specific circRNA, hsa_circ_0049356, in the whole blood and seminal plasma of patients with idiopathic NOA compared to samples derived from healthy individuals. Hsa_circ_0049356 was significantly upregulated in the whole blood and significantly downregulated in the seminal plasma of NOA patients. Furthermore, according to the constructed circ_0049356-miRNA-mRNA interaction networks, this circRNA was found to contribute to the regulation of guanyl-nucleotide exchange factor activity and the regulation of the actin cytoskeleton. Abnormalities in cytoskeleton rearrangement can affect spermatogenesis, while the guanyl-nucleotide exchange factor (GEF) plays a critical role in the differentiation of testicular germ cells. More interestingly, the study of Liu et al. (2020) [44] showed that circRNAs are stable in seminal plasma at room temperature and therefore have the potential to be used as molecular biomarkers. 

Finally, Ji et al. (2021) [46] performed a study to address the question of whether seminal plasma (SP) circRNAs can be used as biomarkers to predict the outcome of microdissection testicular sperm extraction (micro-TESE) in patients with NOA. Micro-TESE is a surgical procedure used for sperm retrieval; however, as the success rate of micro-TESE in NOA patients is not very high and it is an invasive process, it is very important to find accurate biomarkers to screen patients. Using testicular samples from micro-TESE of NOA samples, the researchers found DE circRNAs between patients with successful and unsuccessful sperm retrieval. Moreover, they focused on three of these circRNAs (Table 2) that were verified in SP and testicular tissue samples and showed excellent diagnostic performance in SP in predicting the outcome of micro-TESE. The ceRNA networks that were constructed for these circRNAs also included many genes related to infertility. The authors draw particular attention to the role of the Wnt signaling pathway in the pathogenesis of NOA, which seems to be affected by DE circRNAs. Finally, it was demonstrated that the expression levels of circRNAs were stable in SP at room temperature, indicating that they can be used as biomarkers to screen patients eligible for micro-TESE.

It should be noted that in many of the above studies, OA patients were used as a control group, and they were compared with NOA to identify DE circRNAs. Although an ideal control is considered a testicular sample from individuals with proven fertility or normozoospermic men, it is extremely difficult to obtain such samples, and only one study recruited healthy individuals that were compared with NOA patients [44].

The DE circRNAs with a role in azoospermia identified in the above studies are presented in Table 2. It should be noted that the full list of DE circRNAs was provided only in the publications of Bo et al. (2020) [42] and Ji et al. (2021) [46]; thus, only the verified circRNAs are listed below.

### 3.3. Asthenozoospermia (n = 3)

The second-most studied subtype of male infertility, after azoospermia, is asthenozoospermia. Two of the studies included in the present systematic review used a similar approach, as explained below. More specifically, Manfrevola et al. (2020) [36] characterized the circRNA profile of asthenozoospermic-derived spermatozoa. They identified DE circRNAs between two populations of spermatozoa of asthenozoospermic men that were characterized as “good quality” (A SPZ) and “low quality” (B SPZ) according to their morphological and motility parameters. The DE circRNAs between A and B SPZ that were verified using qRT-PCR are presented in Table 3. DE circRNAs mainly affect pathways that are related to mitochondrial function and sperm motility. Furthermore, the authors constructed ceRNA networks for circUSP54 and found that it affects mitochondrial physiology. Mitochondria are the energy machines of the cell, indicating that deregulation in energy production through circRNAs may affect sperm motility. However, the most interesting part of this study is the fact that the researchers tried to assess the effect of pharmacological treatments aimed at improving the clinical profile of patients (mixture of amino acids, ornithine-citrulline, L-arginine) on circRNA expression before and after treatment. After the treatment period, sperm motility increased from 20% to 40%, and circRNAs that were upregulated in pre-treatment samples decreased their expression levels in post-treatment, similar to the levels observed in normozoospermic men. These findings suggest that the circRNA pattern may fluctuate with a high degree of plasticity and that sperm with altered motility have their own circRNA payload. Manfrevola et al. (2021) [37] took their research a step further by focusing on specific genes that have recently been associated with asthenozoospermia: *CRISP2, CATSPER1,* and *PATE1.* All three mRNAs were significantly reduced in B SPZ compared to A SPZ of asthenozoospermic patients, and then the researchers used bioinformatics tools to construct the ceRNA networks that potentially regulate the expression of these genes. They identified three miRNAs (hsa-miR6721–5p, hsa-miR-138–5p, and hsa-miR-27b) able to target *CATSPER1, PATE1*, and *CRISP2*, respectively. According to the predicted networks, they suggest that the circRNAs circTRIM2, circEPS15, and circRERE, which are also downregulated in B SPZ according to their findings, are potentially involved in the regulation of the above genes. They also tested the effect of the oral amino acid supplementation used in the previous study again. The expression of *CATSPER1* and *PATE1* was significantly restored in B SPZ after the treatment, whereas *CRISP2* expression decreased. Regarding the circRNAs that were downregulated in B SPZ before treatment, all three increased significantly after the pharmacological treatment. These results do not support the regulation of *CRISP2* expression by circRERE, but the authors suggest that another circRNA might be involved (circSEPT10). Thus, although these two studies suggest an intriguing role for circRNAs in sperm motility, they are not definitive about the role of specific circRNAs, and further research is required. It should also be noted that these two studies [36,37] identified DE circRNAs between “bad” and “good” quality SPZ derived from asthenozoospermic men. This may limit the generalizability of these results about circRNAs involved in asthenozoospermia, as it is not clear if the same findings would have been observed from the comparison between asthenozoospermic and normozoospermic men. The DE circRNAs mentioned above are also presented in Table 3.

Gao et al. (2020) [40] performed another type of experimental study aiming to evaluate the role of circBoule RNAs in male infertility as well as their conservation and evolutionary significance. Among other interesting findings, they report that circBoule RNAs are found in mature human sperm and testes and interact with heat shock proteins (HSPs), regulating their levels. HSPs have been implicated in playing a role in male fertility, as spermatozoa are subjected to heat shock after ejaculation and during their transition to the female reproductive tract [50]. Testes and sperm are also generally susceptible to environmental heat stress. Furthermore, Gao et al. (2020) [40] provide evidence that lower levels of the circBoule RNAs circEx3-6 and circEx2-7 are found in semen samples from asthenozoospermic men (Table 3). HSPA2 protein levels were also negatively correlated with circEx3-6 RNA levels in normozoospermic sperm samples but not in asthenozoospermic. As aberrant levels of HSPA2 have been associated in previous studies with human asthenozoospermia [51], the negative correlation between circEx3–6 and HSPA2 and reduced overall levels of circEx3-6 and circEx2-7 in asthenozoospermia suggest that circBoule RNAs may play an important role in human fertility. The authors conclude that circBoule RNAs may have a conserved role in animal sperm development and maturation. 

### 3.4. Oligoasthenozoospermia (n = 1)

Only one study included in the present systematic review explored the role of circRNAs in oligoasthenozoospermia (OAZ). Yue et al. (2022) [39] investigated the circRNA profile of exosomes derived from seminal plasma (SP) of patients with OAZ, as recently, exosomes have been proven to affect all sperm functions required for effective fertilization [52]. Although the authors emphasize that this is a preliminary study, their findings are particularly valuable. They list DE circRNAs between OAZ patients and healthy men, and, according to the circRNA-microRNA-mRNA networks constructed, they suggest that circRNAs exert a negative feedback effect on gene expression regulation that affects sperm motility and the spermatogenesis process. KEGG analysis also revealed several pathways that seem to be involved in the pathophysiology of OAZ (phosphatidylinositol signaling, cell cycle regulation, lysine degradation, etc.). Furthermore, although the exact mechanism of circRNAs in OAZ is not yet understood, as the researchers point out, their differentiated profile between patients and healthy males, as well as their unique properties, support their use as noninvasive biomarkers for the prognosis and diagnosis of OAZ. Interestingly, they observed that though the sperm count was significantly different between normozoospermic men and OAZ patients, no significant differences in the amount of seminal plasma exosomes were observed between the two groups.

The DE circRNAs validated by qRT-PCR in the above-mentioned study are presented in Table 4. 

### 3.5. A Mixed Sample of Infertile Men (n = 1)

The study of Oluwayiose et al. (2022) [41] was the only one included in the present systematic review that used a mixed sample of infertile men and not patients with a specific subtype of male infertility. More specifically, they studied the non-coding RNA profile of seminal plasma (SP) extracellular vesicles (EVs) in a mixed sample of infertile patients characterized as having “poor semen” characteristics if at least one of the three parameters used to evaluate semen quality (motility, morphology, and concentration) was below the WHO reference values. Normozoospermic men were recruited as the control group. Small RNA sequencing (sRNA seq) revealed 57 differentially expressed ncRNA biotypes among men with poor semen quality, of which the most were circRNAs (n = 34) (Table 5). According to circRNA-miRNA-mRNA interactions, the genes targeted were mainly associated with cellular communication and early development. However, it should be noted that all samples used in this study (both infertile men and the control group) were derived from couples undergoing in vitro fertilization. Therefore, this may limit the generalizability of the findings to normozoospermic men with proven fertility status.

### 3.6. Normozoospermia (n = 1)

The study of Chioccarelli et al. (2019) [53] employed a circRNA microarray to investigate the expression patterns of circRNAs in spermatozoa from healthy normozoospermic individuals, differentiating between high- and poor-quality spermatozoa. Semen samples were separated into two populations based on morphology and motility parameters using a Percoll gradient separation. Fraction A (A SPZ) represented spermatozoa of high quality, while fraction B (B SPZ) comprised spermatozoa of very poor quality. The study identified a total of 148 differentially expressed circRNAs (91 up-regulated and 57 down-regulated circRNAs in fraction B compared to fraction A). The circRNAs that exhibited differential expression were subsequently validated using quantitative PCR, and the validated circRNAs are presented in Table 6. The authors also explored the interaction networks of the DE circRNAs. Notably, these circRNAs exhibited a distinctive pattern, as evidenced by two DE circRNAs displaying tethering activity towards the same set of miRNAs, while more than two DE circRNAs exhibited tethering activity towards a specific miRNA. These observations prompt the hypothesis that circRNAs derived from SPZs may function through functional clusters to ensure successful fertilization. Additionally, the authors investigated the subcellular localization of the validated circRNAs that were differentially expressed between B and A SPZ. This examination was conducted using enriched preparations of the spermatozoa head and tail. Interestingly, this experiment revealed that circRNAs exhibited a higher expression preference in the SPZ head preparation, indicating their potential transmission from the SPZ to the oocyte during fertilization.

It should be noted that in all the above tables, derived genes were found according to the Circbank [54] and CircAtlas databases [55].

## 4. Discussion

Recent advances in molecular technologies, as well as the wide use of genome sequencing and transcriptomic analyses, have enhanced our understanding of male infertility. CircRNAs are a new class of RNA molecules with cell- and tissue-specific expression and are conserved among eukaryotes [56]. Furthermore, they contribute to the regulation of many cellular processes and are released in body fluids, where they are highly stable. Therefore, the use of circRNAs as novel biomarkers shows great promise and may eventually have a significant impact on clinical practice for male infertility diagnosis and prognosis. Their study could also provide a new perspective on our understanding of the molecular mechanisms and pathogenesis of male infertility. Thus, we reviewed studies investigating the role of circRNAs in male infertility and summarized DE circRNAs from all published studies in humans, evaluating the processes and pathways that are affected, mainly through circRNA-miRNA interactions, as well as the diagnostic performance of circRNAs. 

**Figure 6:**
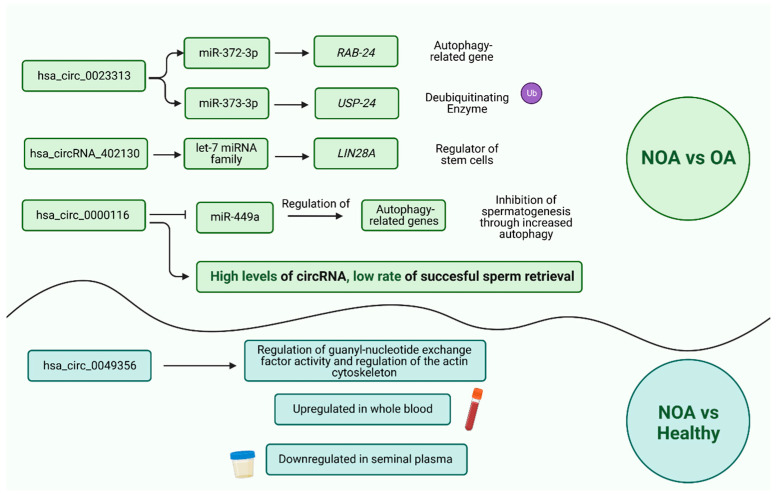
CircRNAs with a role in NOA pathogenesis. Image created with Biorender.com (accessed on 26 June 2023).

In the present systematic review, 13 articles were included in the final sample analyzed. Azoospermia is the most well-studied subtype of male infertility regarding circRNAs, probably because it is the most well-defined. Therefore, more than half of the studies were about azoospermia. Most studies focus on the role of circRNAs in NOA. The circRNAs reported in the studies included in the present systematic review to be involved in NOA are presented in Figure 6. Furthermore, the above studies confirm the continuous crosstalk of circRNAs and microRNAs. Usually, circRNAs act as miRNA inhibitors or sponges, and with this mechanism, they participate in regulating mRNA expression in various human diseases, including male infertility. As observed above, many cellular processes and pathways are deregulated by circRNA-miRNA interactions, leading to NOA pathogenesis. The development of biomarkers for screening patients undergoing micro-TESE was also investigated in NOA patients in the publication of Ji et al. (2021) [46].

**Figure 7:**
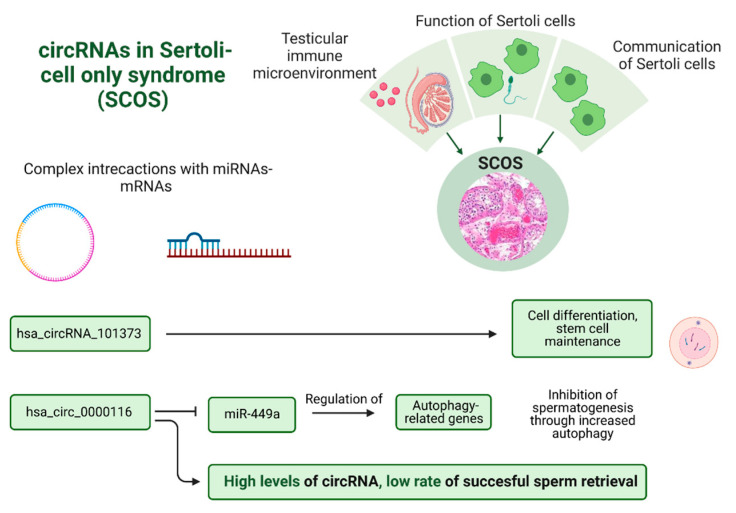
CircRNAs associated with Sertoli cell-only syndrome and pathways that are deregulated. Image created with biorender.com (accessed on 26 June 2023).

Finally, particular subtypes of azoospermia have also received attention, and specific circRNAs have been associated with them. For example, the main circRNAs associated with SCOS and the pathways deregulated by them are presented in Figure 7. 

It should be noted that Figure 6 and Figure 7 present circRNAs that were further investigated with extra experiments in the articles included in the present systematic review. These circRNAs were specifically selected based on the availability of more information provided by the above articles regarding their potential role, interactions, or mechanism of action related to male infertility. As a result, these figures serve as a summary of the main findings derived from these experiments described in the articles included in the present systematic review, focusing on these selected circRNAs.

Of the above circRNAs, hsa_circRNA_101373 has been reported in a study performed in rats and is associated with light-induced ovarian dysfunction [57]. Some circRNAs have also been implicated to have a role in cancer, e.g., hsa_circ_0000277 is involved in esophageal cancer [58,59], whereas hsa_circ_0007773 has been indicated to inhibit the progression of hepatocellular carcinoma [60]. Interestingly, the other circRNAs have not been associated with specific diseases or with the regulation of specific cellular processes. 

Furthermore, some studies included in the present systematic review focused on the role of circRNAs in asthenozoospermia. Two studies [36,37] report that some of the DE circRNAs identified (circUSP54) are mainly associated with mitochondrial physiology and energy production. These results are consistent with previous studies that associate decreased sperm motility with defects in mitochondria, the powerhouse of the cell [61,62,63]. Furthermore, the most important finding of these studies is that the circRNA expression pattern is characterized by high plasticity. circBoule RNA levels also exert lower levels in asthenozoospermic men, and these circRNAs play a role in heat stress-induced fertility decline [40]. 

Regarding other subtypes of male infertility, only a preliminary study about circRNAs in OAZ was found [39]. This was particularly focused on the circRNA profile of exosomes derived from seminal plasma. In this study, the important role of circRNA-microRNA-mRNA networks in male infertility is again highlighted, as in previous cases, but notably, the authors suggest that exosome-derived circRNAs could be used as potential noninvasive biomarkers. Of the identified DE circRNAs, hsa_circ_0003823 is associated with esophageal cancer, according to previous research [64]. 

One more study included in the present systematic review was performed in a mixed sample of infertile men and supported the use of circRNAs found in seminal plasma extracellular vesicles as biomarkers of male infertility [41]. In general, the diagnostic potential of circRNAs is supported by many studies regarding various diseases, as they exert high tissue specificity, are stable in body fluids, and are easily detected [65]. 

Finally, one study [53] was conducted on normozoospermic men, differentiating spermatozoa into two populations based on the Percoll gradient. They identified DE circRNAs between spermatozoa of high and poor quality, and furthermore, they indicate that circRNAs may be transmitted to the oocyte during fertilization. Such studies may shed light on cases of normozoospermic men that are unable to achieve pregnancy and require further research, highlighting that complex mechanisms are involved in male infertility.

It should also be noted that no common DE circRNAs were observed between the different studies or the different subtypes of male infertility. This may be due to the variability of the methods used in the above studies and draws attention to issues of comparability between sequencing and array-based technologies. In general, array-based methods are usually considered more prone to false positives, especially when combined with limited sample sizes [41], and thus, the results of these studies should be interpreted with caution. 

Based on the above and taking into consideration the methodology we followed, the present systematic review has some significant limitations. First, studies on animals (both experimental animals and livestock) were excluded because our main goal was to report DE circRNAs that have been identified and validated in humans and thus have a greater potential for clinical applications. However, with this approach, some information may have been overlooked. Another limitation of the present systematic review is that we were unable to retrieve every article. Specifically, despite our best efforts, we were unable to retrieve one article [66]. This source may refer to an abstract of a conference presentation, which would be excluded from our systematic review according to our pre-defined exclusion criteria. However, since we were unable to find this article, there is a possibility that relevant information may have been overlooked. Furthermore, because we removed studies that were not published in English, we may have overlooked significant papers that were published in other languages. Another limitation of the present systematic review is that it focused solely on articles reporting DE circRNAs between men with varying fertility statuses (fertile or infertile). However, there are other four studies that, although not reporting DE circRNAs, offer valuable insights. More specifically, Dong et al. (2016) [67] conducted a comprehensive profiling of circRNAs in the testicular tissues of healthy men and identified a large number of novel circRNAs. They further investigated the expression patterns and characteristics of these circRNAs. The study found that circRNAs derived from the human testis exhibited tissue-specific expression and were enriched in specific biological processes and pathways. Additionally, the researchers identified certain circRNAs that were detectable in the seminal plasma, indicating their potential as noninvasive biomarkers for male reproductive health. Similarly, Ragusa et al. (2019) [68] investigated the presence and interaction of a specific circRNA, circNAPEPLD, in both human and murine spermatozoa. The study revealed the expression of circNAPEPLD in both human and murine spermatozoa through various experimental techniques, and the researchers found that circNAPEPLD physically interacts with miRNAs present in oocytes, suggesting a potential regulatory role during fertilization. These findings may shed light on the molecular mechanisms underlying reproductive processes and provide a basis for further investigation into the functional significance of circRNAs in fertility and fertilization. Tang et al. (2020) [69] performed another type of study as they aimed to understand the regulatory mechanisms involved in circRNA biogenesis and the impact of m^6^A modification on this process. More specifically, they identified specific m^6^A-modified regions within precursor messenger RNAs (pre-mRNAs) that contribute to circRNA formation. By manipulating the m^6^A machinery, they were able to modulate circRNA abundance, proving that these modifications play a crucial role in circRNA biogenesis in male germ cells. Lastly, Liu et al. (2020) [70] investigated the effects of l-carnitine on the expression of miRNAs in the spermatozoa of individuals with asthenozoospermia. The study demonstrated that l-carnitine treatment influences the expression levels of specific miRNAs. By employing high-throughput sequencing and bioinformatics analysis, they identified the miRNAs that are differentially expressed upon l-carnitine treatment, and additionally, they explored the regulatory network involving these miRNAs and their associated molecules. Among other molecules, they report interactions with specific circRNAs. Thus, all the above may have led to the small number of studies included in the present systematic review jeopardizing solid conclusions, but in general, it is notable that this review has identified surprisingly little research conducted to date about circRNAs and male infertility. 

Several limitations also arose from the existing studies included in this systematic review. One is that some studies do not present the full list of differentially expressed circRNAs, preventing the identification of common circRNAs between studies or circRNAs that are deregulated in many subtypes of male infertility. Second, the studies developed very different experimental designs and used different technologies, such as the sequencing- and array-based approaches mentioned above, preventing any comparative or cross-analysis. Third, one limitation is the variability in the definitions of differential expression used among the included studies. The studies included employed diverse fold change thresholds to determine differential expression of circRNAs. This variability in defining differential expression may introduce discrepancies and hinder direct comparison and interpretation of the results. Therefore, future research may benefit from establishing standardized guidelines for defining differential expression of circRNAs to enhance comparability and reproducibility in the field of male infertility. Fourth, some of the included studies used normozoospermic males as controls based on seminogram results rather than those confirmed fertile by previous pregnancy outcomes. For example, Oluwayiose et al. (2022) [41] used as a control group normozoospermic men recruited from couples undergoing IVF. This can result in inconsistencies and limit the generalizability of findings. Furthermore, another limitation of this systematic review is the incomplete availability of information regarding both the mapping and circRNA calling processes in most of the included studies. Mapping involves aligning sequencing reads to a reference genome, while circRNA calling focuses on specifically identifying circular RNAs from the mapped reads. The mapping method and circRNA calling algorithms employed can significantly influence the identification and quantification of circRNAs. However, due to the lack of detailed information in some studies, it is challenging to assess the specific mapping methods and circRNA calling algorithms utilized, potentially introducing variability in circRNA detection and expression quantification. The variation in these methods across studies could impact the comparability and interpretation of the findings. Finally, the main limitation of most studies included is their small sample sizes. This can lead to unreliable results, and thus, validation in larger cohorts is required.

Despite the limitations described above, our study has key strengths. To our knowledge, this is the first systematic review investigating published research linking circRNAs to male fertility and summarizing the main findings of the existing literature. Furthermore, the use of PRISMA guidelines and the developed search strategy were intended to be as inclusive as possible by expanding the search to two renowned databases, such as PubMed and Web of Science. Therefore, we believe that this systematic review will be of particular interest to the life science community and clinical doctors engaged in the field of infertility. The list of DE circRNAs reported here will also help biologists unravel the role of circRNAs in male infertility and pave the way for future studies. 

Regarding future research, compared with other noncoding RNAs, such as lncRNAs and miRNAs, studies of circRNAs in male infertility are just at the beginning. This systematic review points out a huge knowledge gap on the role of circRNAs in subtypes of male infertility other than azoospermia. There are no studies about circRNAs and specific sperm defects, such as teratozoospermia, oligozoospermia, or their combination; therefore, future studies should focus on these directions. In general, most of the circRNAs identified as deregulated have not been associated in previous studies with other diseases or molecular mechanisms, which implies that more research is needed to explore the exact biological functions of circRNAs and their role. Third, most of the interactions between circRNAs and miRNAs reported in the identified studies were predicted using bioinformatics models; therefore, more experimental studies are needed to investigate specific interactions and the networks of circRNAs. For diagnostic applications, more studies should also focus on the sensitivity and specificity of circRNAs applied to male infertility subtypes and evaluate their diagnostic value. Furthermore, existing studies in humans about circRNAs are mainly single experiments with a small number of subjects, which undermines the robustness of the results. Thus, it is essential to perform studies that use a large number of individuals or samples to validate previous findings or conduct new experiments focusing on circRNAs. Collaboration between institutions or research centers would be beneficial in this approach, but unfortunately, such collaborations have not been common in the studies carried out to date. Finally, some of the studies identified in the present systematic review suggest that spermatozoa are cells with unique epigenetic signatures characterized by unique and differential quantitative and qualitative fingerprints of circRNAs [36]. This result is intriguing and raises questions about the real effectiveness of IVF techniques, such as the intracytoplasmic sperm injection (ICSI) procedure, which has been considered a useful approach to selecting good-quality spermatozoa for fertilization. It also raises the question of whether this unique epigenetic signature has an impact on the offspring, as the use of IVF is on the rise. Thus, further research is also required in this direction.

## 5. Conclusions

In conclusion, this is the first systematic review to evaluate all published literature exploring the role of circRNAs in male infertility, with a particular emphasis on studies of human subjects. In this review, we attempted to provide a comprehensive summary of all DE circRNAs that play a role in male infertility. These circRNAs exert their function mainly as miRNA sponges, regulating the expression level of target mRNAs. In this way, multiple cellular signaling pathways and processes, such as meiosis, stem cell pluripotency, autophagy, etc., are deregulated, contributing to the pathogenesis of male infertility. Furthermore, given their characteristics of high stability and specificity, circRNAs are expected to be potential novel biomarkers for the diagnosis of male infertility, with a promising role in predicting sperm retrieval in patients undergoing micro-TESE. Finally, it seems that the study of circRNAs in male infertility is a new field, and there is much more to explore, as except for azoospermia, information about other circRNAs and other subtypes of male infertility is missing.

## Figures and Tables

**Table 1:** Inclusion and exclusion criteria for the selection of articles included in the present systematic review.

Inclusion Criteria	Exclusion Criteria
Studies in the English language	Studies not in the English language
Human male adult participants	Cell lines, animal, and plant studies
Experimental studies reporting DE circRNAs and aiming at associating circRNAs with male infertility	Reviews, meta-analyses, books, book chapters, letters, commentary articles, editorials, replies, abstracts of conference presentations, etc.
	Studies on other types of noncoding RNAs (miRNAs, lncRNAs, etc.) except for circRNAs

**Table 2:** CircRNAs with a potential role in azoospermia according to the above publications and study characteristics; FC: fold change; UP: upregulated; DOWN: downregulated.

Reference	Year of Publication	Comparison	Samples	Methodology	Definition of DE	CircRNAs	Derived Genes	Change of Expression
Ge et al. [43]	2019	NOA vs. OA	Testis	Microarray, qRT-PCR	FC > 2 and *p*-value < 0.05	hsa_circ_0058058 (hsa_circATIC_002)	*ATIC*	UP
						hsa_circ_0008045 (hsa_circMGLL_009)	*MGLL*	UP
						hsa_circ_0023313 (hsa_circPPFIA1_001)	*PPFIA1*	UP
						hsa_circ_0061817 (hsa_circC2CD2_004)	*C2CD2*	DOWN
						hsa_circ_0002023 (hsa_circCDC25A_005)	*CDC25A*	DOWN
						hsa_circ_0008533 (hsa_circCLTCL1_021)	*CLTCL1*	DOWN
Bo et al. [42]	2020	NOA vs. OA	Testis	Microarray, qRT-PCR	FC ≥ 1.5 and *p*-value < 0.05	hsa_circRNA_402130	NA	DOWN
						hsa_circRNA_072697	NA	UP
						hsa_circRNA_030050	NA	UP
						hsa_circRNA_100812	NA	DOWN
						hsa_circRNA_406168	NA	DOWN
Zhang et al. [45]	2022	NOA vs. OA	Testis	Array, qRT-PCR	FC > 2 and *p*-value < 0.05	hsa_ circ_0137890	NA	DOWN
						hsa_circ_0136298 (hsa_circCSMD1_051)	*CSMD1*	DOWN
						hsa_circ_0007273 (hsa_circRAB11FIP5_002)	*RAB11FIP5*	DOWN
Zhu et al. [47]	2021	SCOS (NOA) vs. OA	Testis	Microarray, qRT-PCR	FC > 2 and *p*-value < 0.05	hsa_circRNA_101222	ΝA	DOWN
						hsa_circRNA_001387	ΝA	DOWN
						hsa_circRNA_103486	ΝA	DOWN
						hsa_circRNA_001153	ΝA	UP
						hsa_circRNA_101373	ΝA	UP
						hsa_circRNA_103864	ΝA	UP
Lv et al. [38]	2020	NOA vs. OA	Testis	qRT-PCR	*p*-value < 0.05	hsa_circ_0000116 (hsa_circMAN1A2_006)	*MAN1A2*	UP
		SCOS vs. HS and OA						UP
Liu et al. [44]	2020	NOA vs. Healthy	Whole Blood	qRT-PCR	*p*-value < 0.05	hsa_circ_0049356 (hsa_circCARM1_014)	*CARM1*	UP
			SP					DOWN
Ji et al. [46]	2021	NOA with successful sperm retrieval vs. NOA with non-successful sperm retrieval	SP and Testis	RNA sequencing, qRT-PCR	|log_2_ (FC)| > 1 and *q*-value < 0.05	hsa_circ_0000277 (hsa_circPDE3B_001)	*PDE3B*	UP
						hsa_circ_0060394 (hsa_circZHX3_008)	*ZHX3*	UP
						hsa_circ_0007773 (hsa_circFAM114A2_005)	*FAM114A2*	UP

**Table 3:** CircRNAs with a potential role in asthenozoospermia according to the above publications and study characteristics; FC: fold change; UP: upregulated; DOWN: downregulated.

Reference	Year of Publication	Comparison	Samples	Methodology	Definition of DE	CircRNAs	Derived Genes	Change of Expression
Manfrevola et al. [36]	2020	B vs. A SPZ (“Bad” vs. “Good quality” SPZ from Asthenozoospermic)	Semen	Microarray, qRT-PCR	FC ≥ 1.5 and *p*-value ≤ 0.05	circMCC	*MCC*	UP
						circPAPPA2	*PAPPA2*	UP
						circSLC25A26	*SLC25A26*	UP
						circCANX	*CANX*	UP
						circDDX17	*DDX17*	UP
						circHDAC3	*HDAC3*	UP
						circHDAC3	*HDAC3*	UP
						circDYNC1H1	*DYNC1H1*	UP
						circFABP6	*FABP6*	UP
						circTADA2A	*TADA2A*	DOWN
						circPEX1	*PEX1*	DOWN
						circATF	*ATF*	DOWN
						circUSP54	*USP54*	DOWN
						circCLSPN	*CLSPN*	DOWN
						circTRMT2B	*TRMT2B*	DOWN
						circCIT	*CIT*	DOWN
						circEPS15	*EPS15*	DOWN
						circPTBP3	*PTBP3*	DOWN
Manfrevola et al. [37]	2021	B vs. A SPZ (“Bad” vs. “Good quality” SPZ from Asthenozoospermic)	Semen	qRT-PCR	*p*-value < 0.05	circTRIM2	*TRIM2*	DOWN
						circEPS15	*EPS15*	DOWN
						circRERE	*RERE*	DOWN
						circSEPT10	*SEPT10*	DOWN
Gao et al. [40]	2020	Asthenozoospermia vs. Normozoospermia	Semen	qRT-PCR	*p*-value < 0.05	circBoule RNAs (circEx3–6 and circEx2–7)	*BOULE*	DOWN

**Table 4:** CircRNAs with a potential role in OAZ according to the above publication and study characteristics; FC: fold change; UP: upregulated; DOWN: downregulated.

Reference	Year of Publication	Comparison	Samples	Methodology	Definition of DE	CircRNAs	Derived Genes	Change of Expression
Yue et al. [39]	2022	OAZ vs. Healthy	Exosomes (SP)	RNA sequencing, qRT-PCR	FC > 2 and *p*-value < 0.05	chr3:132050491–132051188 + (novel)	NA	UP
						hsa_circ_0005584 (hsa_circMBD2_003)	*MBD2*	UP
						hsa_circ_0003823 (hsa_circCEP70_005)	*CEP70*	UP
						hsa_circ_0125759 (hsa_circNEK1_026)	*NEK1*	UP
						hsa_circ_0009142 (hsa_circCAP1_006)	*CAP1*	UP
						chr12:130827535–130846146 + (novel)	NA	DOWN
						hsa_circ_0002452 (hsa_circPUM1_023)	*PUM1*	DOWN

**Table 5:** CircRNAs with a potential role in male infertility according to the above publication using a mixed sample of infertile men and study characteristics; FC: fold change; FDR: False Discovery Rate; UP: upregulated; DOWN: downregulated.

Reference	Year of Publication	Comparison	Samples	Methodology	Definition of DE	CircRNAs	Derived Genes	Change of Expression
Oluwayiose et al. [41]	2022	“Poor semen” vs. Nomozoospermic	EVs (SP)	sRNA seq	FC ≥ 1.5 and FDR < 0.05	hsa_circ_0009013 (hsa_circACAP2_004)	*ACAP2*	UP
						hsa_circ_0123184 (hsa_circACAP2_003)	*ACAP2*	UP
						hsa_circ_0114168 (hsa_circZZZ3_001)	*ZZZ3*	DOWN
						hsa_circ_0139507 (hsa_circRAB40A_003)	*RAB40A*	UP
						hsa_circ_0139505 (hsa_circLL0XNC01–250H12.3_001)	*ENSG00000234405* (lnc-TCEAL4–2)	UP
						hsa_circ_0139508 (hsa_circTCONS_00017354_001)	*ENSG00000264869* (lnc-CCDC102B-5)	UP
						hsa_circ_0001488 (hsa_circPDE4D_012)	*PDE4D*	DOWN
						hsa_circ_0005447 (hsa_circXXYLT1_007)	*XXYLT1*	UP
						hsa_circ_0135261 (hsa_circVPS13B_041)	*VPS13B*	UP
						hsa_circ_0103367 (hsa_circC15orf41_007)	*C15orf41*	DOWN
						hsa_circ_0126706 (hsa_circ_chr4_00008)	Intergenic	UP
						hsa_circ_0009684 (hsa_circUBE4B_002)	*UBE4B*	DOWN
						hsa_circ_0096701 (hsa_circGRM5_002)	*GRM5*	UP
						hsa_circ_0119752 (hsa_circMEMO1_001)	*MEMO1*	DOWN
						hsa_circ_0130725 (hsa_circ_chr6_00085)	Intergenic	UP
						hsa_circ_0132863 (hsa_circLHFPL3_002)	*LHFPL3*	DOWN
						hsa_circ_0056159 (hsa_circIL1RN_001)	*IL1RN*	DOWN
						hsa_circ_0128521 (hsa_circODZ2_006)	*ODZ2*	UP
						hsa_circ_0063775 (hsa_circLOC150381_001)	*LOC150381*	UP
						hsa_circ_0098728 (hsa_circDIP2B_002)	*DIP2B*	DOWN
						hsa_circ_0120217 (hsa_circPPP1R21_035)	*PPP1R21*	DOWN
						hsa_circ_0115951 (hsa_circMORC3_039)	*MORC3*	UP
						hsa_circ_0036948 (hsa_circSLCO3A1_003)	*SLCO3A1*	UP
						hsa_circ_0123091 (hsa_circCLDN1_001)	*CLDN1*	UP
						hsa_circ_0136453 (hsa_circRP11–473J6.1_001)	*RP11–473J6.1*	DOWN
						hsa_circ_0004923 (hsa_circSH3RF3_001)	*SH3RF3*	DOWN
						hsa_circ_0108420 (hsa_circPIK3C3_050)	*PIK3C3*	UP
						hsa_circ_0129336 (hsa_circADAMTS6_048)	*ADAMTS6*	DOWN
						hsa_circ_0132209 (hsa_circRIMS1_009)	*RIMS1*	UP
						hsa_circ_0139547 (hsa_circTMEM164_001)	*TMEM164*	UP
						hsa_circ_0113676 (hsa_circPRKAA2_001)	*PRKAA2*	DOWN
						hsa_circ_0100429 (hsa_circFREM2_001)	*FREM2*	DOWN
						hsa_circ_0024724 (hsa_circGRAMD1B_001)	*GRAMD1B*	UP
						hsa_circ_0113744 (hsa_circTCONS_00000081_003)	*LINC01748*	DOWN

**Table 6:** CircRNAs with a potential role in male infertility according to the above publication using two different SPZ populations in semen samples from healthy normozoospermic men; FC: fold change; UP: upregulated; DOWN: downregulated.

Reference	Year of Publication	Comparison	Samples	Methodology	Definition of DE	CircRNAs	Derived Genes	Change of Expression
Chioccarelli et al. [53]	2019	B vs. A SPZ (“poor quality” vs. “high quality”)	Semen	Microarray	FC > 1.5 and *p*-value < 0.05	circANKLE2	*ANKLE2*	UP
						circMTND5	*MTND5*	UP
						circMLLT3	*MLLT3*	UP
						circSEC24	*SEC24*	UP
						circUBA2	*UBA2*	UP
						circSENP6	*SENP6*	UP
						circRASA3	*RASA3*	UP
						circJA760600	*JA760600*	UP
						circGPBP1L1	*GPBP1L1*	UP
						circGRB10	*GRB10*	UP
						circWDR66	*WDR66*	UP
						circEIF2C2	*EIF2C2*	UP
						circATF7IP	*ATF7IP*	UP
						circPTTG1IP	*PTTG1IP*	DOWN
						circKIF2C	*KIF2C*	DOWN
						circVMP1	*VMP1*	DOWN
						circHACE1	*HACE1*	DOWN
						circZFAT	*ZFAT*	DOWN
						circLZIC	*LZIC*	DOWN
						circCNOT6L	*CNOT6L*	DOWN
						circGUSBP1	*GUSBP1*	DOWN
						circL3MBTL4	*L3MBTL4*	DOWN
						circDNAJB6	*DNAJB6*	DOWN
						circZMYND11	*ZMYND11*	DOWN
						circZNF148	*ZNF148*	DOWN
						circUBXN7	*UBXN7*	DOWN

## Data Availability

Not applicable.
